# CircFOXP1/FOXP1 promotes osteogenic differentiation in adipose‐derived mesenchymal stem cells and bone regeneration in osteoporosis via miR‐33a‐5p

**DOI:** 10.1111/jcmm.15792

**Published:** 2020-09-30

**Authors:** Wanxiang Shen, Bin Sun, Chenghong Zhou, Wenyi Ming, Shaohua Zhang, Xudong Wu

**Affiliations:** ^1^ Department of Orthopaedics Zhoushan Hospital of Traditional Chinese Medicine Affiliated to Zhejiang Chinese Medical University Zhejiang China; ^2^ Inspection Division Zhoushan Hospital of Traditional Chinese Medicine Affiliated to Zhejiang Chinese Medical University Zhejiang China

**Keywords:** bone regeneration, circular RNAs (circRNAs), human adipose‐derived mesenchymal stem cells (hASCs), miR‐33a‐5p, osteogenic differentiation, osteoporosis (OP)

## Abstract

Osteoporosis (OP) is defined by bone mass loss and structural bone deterioration. Currently, there are no effective therapies for OP treatment. Circular RNAs (circRNAs) have been reported to have an important function in stem cell osteogenesis and to be associated with OP. Most circRNA roles in OP remain unclear. In the present study, we employed circRNA microarray to investigate circRNA expression patterns in OP and non‐OP patient bone tissues. The circRNA‐miRNA‐mRNA interaction was predicted using bioinformatic analysis and confirmed by RNA FISH, RIP and dual‐luciferase reporter assays. ARS and ALP staining was used to detect the degree of osteogenic differentiation in human adipose‐derived mesenchymal stem cells (hASCs) in vitro. In vivo osteogenesis in hASCs encapsulated in collagen‐based hydrogels was tested with heterotopic bone formation assay in nude mice. Our research found that circFOXP1 was significantly down‐regulated in OP patient bone tissues and functioned like a miRNA sponge targeting miR‐33a‐5p to increase FOXP1 expression. In vivo and in vitro analyses showed that circFOXP1 enhances hASC osteogenesis by sponging miR‐33a‐5p. Conversely, miR‐33a‐5p inhibits osteogenesis by targeting FOXP1 3′‐UTR and down‐regulating FOXP1 expression. These results determined that circFOXP1 binding to miR‐33a‐5p promotes hASC osteogenic differentiation by targeting FOXP1. Therefore, circFOXP7ay prevent OP and can be used as a candidate OP therapeutic target.

## 1 INTRODUCTION

Osteoporosis (OP) is a common bone disease identified by low bone mass and defective bone structure, leading to bone fragility or high risk of fracture.^1^ It develops as an age‐related bone malady and is usually followed by menopause in women and occurs later in life among men. OP seriously affects patient life quality and leads to considerable financial burden.^2^ Some progress has been made in preventing OP and developing therapeutic methods for reducing fracture risk. However, currently available treatments do little to cure OP completely.^3^, ^4^ With the rapid development of cell‐based therapy, mesenchymal stem cells (MSCs) have become the focus of new treatments for osteoporosis. Recently, adipose‐derived stem cells (ASCs) have become a popular cell source of MSCs because of their minimally invasive acquisition, greater abundance and high production.^5^ Ye et al found that ASCs increased bone mineral density and new bone formation in an OVX‐induced osteoporotic rabbit model.^6^ However, lots of molecules and signalling pathways involved in osteogenic differentiation of ASCs remain unknown, and considerable research is needed to reveal the associated mechanisms and to induce ACS osteogenesis effectively and safely for manipulating ASC‐based cell therapy in osteoporosis treatment. In recent years, more studies have demonstrated that circular RNAs (circRNAs) have a critical function in regulating various signalling pathways related to osteogenesis.^7^ Therefore, circRNAs are regarded as a new focus point in bone research. The circRNAs belong to a particular class of noncoding RNA with a covalently closed loop structure. They are found in eukaryotic transcriptome and play various roles in multiple physiological and pathological processes.^8^, ^9^ The essential physiological circRNA functions include protein translation templates, miRNA sponges and parental expression regulation.^10^, ^11^ Recent studies have revealed that mRNAs, lncRNAs, circRNAs and miRNAs are abnormally expressed in peripheral blood lymphocytes in postmenopausal OP patients and are correlated with disease occurrence.^12^ Several circRNAs have been identified to be essential in regulating stem cell osteogenic differentiation.^13^, ^14^ For instance, circPOMT1 and circMCM3AP inhibit human adipose‐derived stem cell (hASC) osteogenic differentiation by targeting hsa‐miR‐6881‐3p via the bone morphogenetic protein (BMP) signalling pathway.^15^ Moreover, circRNA runt‐related transcription factor 2 (circRUNX2) is down‐regulated in OP patient bone tissues and enhances human bone mesenchymal stem cell (hBMSC) osteogenic differentiation by sponging miR‐203 and regulating the expression of RUNX2 and osteocalcin (OCN, as a late marker for bone formation).^16^ As a master regulator for osteogenesis, Runx2 plays a key role in co‐ordinating multiple signalling pathways involved in osteoblast differentiation.^17^, ^18^ These studies suggest that circRNAs might take part in the osteogenesis process.

Studies have revealed that multiple microRNAs (miRNAs) play a pivotal role in bone homeostasis such as MSC differentiation, survivability and apoptosis, adipogenesis and osteogenesis.^19^, ^20^ Previous studies have shown that miR‐21 promoted osteogenic differentiation of MSCs derived from human umbilical cord,^21^ but enhanced adipogenic differentiation in ASCs,^22^ suggesting that the same miRNA may play different roles in different MSCs. It is known that in bone tissue, miR‐33a plays a role in osteosarcoma chemoresistance by down‐regulating TWIST^23^ and in osteoblast differentiation after mechanical stimulations.^24^, ^25^ Furthermore, miR‐33a, which is the only one miR‐33 isoform, is expressed in mice and conserved in humans. Human miR‐33a has two subtypes, miR‐33a‐3p and miR‐33a‐5p, which correspond to miR‐33‐3p and miR‐33‐5p in mice, respectively. The miR‐33a family (3p and 5p) has recently been found to be a possible modulator of YAP/TAZ during hMSCs osteoblast differentiation.^26^ However, its role in osteogenic differentiation of ASCs still needs to be identified.

The present study evaluated circRNA expression profiles in OP and non‐OP patients using microarray analysis and identified 4,972 differentially expressed circRNAs. Among them, circRNA forkhead box P1 (FOXP1) expression levels were significantly down‐regulated in OP patients compared to the expression levels in non‐OP patients. As a transcriptional factor, FOXP1 controls several cell differentiation pathways, such as embryonic stem cell pluripotency,^27^ T‐ and B‐cell development,^28^, ^29^ lung epithelial cell fate determination ^30^ and MSC differentiation.^31^ CircFOXP1 has been demonstrated to play a key role in MSC fate decision‐making processes via miRNA inhibition.^32^ In this study, the role of circFOXP1 was investigated in hASC osteogenic differentiation and bone regeneration. It was discovered that circFOXP1 might act like an miR‐33a‐5p sponge to up‐regulate FOXP1 expression and consequently promote osteogenesis. These findings enhance the current understanding of circRNA and miRNA function in osteogenesis. In addition, circRNA might be a key therapeutic target in OP patients.

## 2 MATERIALS AND METHODS

### 2.1 Clinical samples

Samples of trabecular bone were acquired from the femoral trochanteric region located far from the periarticular bone in OP and non‐OP patients undergoing hip arthroplasty for a fractured femoral neck. A total of 20 OP specimens were collected from ten females (age range: 60‐87, average age: 73) and ten males (age range: 55‐83, average age: 71). A total of 20 non‐OP specimens from ten females (age range: 56‐84, average age: 69) and ten males (age range: 52‐85, average age: 70) who were suffering from external traumatic fracture were collected for the control group. Non‐OP patients were known to have not suffered from any chronic condition or disease that may have affected their skeletal tissue. Before enrolling in the study, none of the patients have experienced any medical therapy that affected their mineral metabolism or bone tissue. The Ethics Committee of Zhoushan Hospital of Traditional Chinese Medicine Affiliated to Zhejiang Chinese Medical University has approved the research protocol, and each patient signed the informed written consent form prior to their participation in this research.

### 2.2 CircRNA microarray analysis

Sample preparation and microarray hybridization were performed following a standard protocol (Arraystar, Rockville, MD, USA). Briefly, in order to eliminate linear RNAs and improve circRNAs, total RNA was digested with RNAse R (Epicentre, Madison, WI, USA). The improved circRNAs were then expanded and transcribed into fluorescent cRNAs using a random priming method with Arraystar Super RNA Labelling Kit (Arraystar). Labelled cRNAs were hybridized onto the Arraystar Human circRNA Array V1.0. Finally, the array was scanned using the Agilent Scanner G2505C and the resulting array images were analysed.

### 2.3 QRT‐PCR analysis

Total RNA and miRNA samples were collected from cultured cells using the Trizol reagent (Invitrogen, Karlsruhe, Germany) and mirVana miRNA isolation kit (Ambion, Austin, TX, USA) based on manufacturer instructions. Tissue Tearor (Biospec Products, Bartlesville, OK, USA) was used to disrupt samples for RNA isolation from osteo tissues. NanoDrop ND‐1000 spectrophotometer (NanoDrop Technologies, Wilmington, DE, USA) was used to verify RNA concentration and quality. Subsequently, cDNA was composed using a Prime Script RT reagent kit (Takara, Beijing, China). SYBR Premix Ex Taq (Takara) was used to perform qPCR analysis in an ABI PRISM 7500 Sequence Detection System (Life Technologies, Grand Island, NY, USA). U6 snRNA was utilized as an endogenous miRNA control, whereas circRNA levels were normalized using GAPDH. Relative gene expression levels were computed using the 2^−ΔΔCt^ method. Primers used for RT‐PCR are included in Table 1.

**TABLE 1:** Primers used in real‐time RT‐PCR analysis

Primer	Sequence (5′‐3′)
has_circ_0002544‐ F	TCTGCCGCTTATCTTCAGGA
has_circ_0002544‐ R	TCAAATGTGGTCTCTGGATTCTG
has_circ_0004834‐ F	ATGCATCAGTTCCATCGAGC
has_circ_0004834‐ R	ATCCCGACTAAGTTTCACAAGT
has_circ_0003457‐ F	GGTCCTGAAGATGATGCTGC
has_circ_0003457‐ R	AGAAGAGAGGGCCAGTTGTG
has_circ_0001921‐ F	GCAACATCCGGGAGTTTGAG
has_circ_0001921‐ R	GTTGGCTAGCTCACTCTCCA
has_circ_0001538‐ F	AGTGATGGCTCTTCTCTGGA
has_circ_0001538‐ R	GGCTGATGTTGGTTGTCGTT
has_circ_0000976‐ F	GAGATGCTGGAGATCGTGC
has_circ_0000976‐ R	CTTGCTGTTCTGTTTGCCCA
has_circ_0004599‐ F	CCAAGATGACCCAACTCCCT
has_circ_0004599‐ R	TGGGTCAGAACAGGTAGCAT
has_circ_0001320‐ F	AAGTGGCCAGGCTGTGAA
has_circ_0001320‐ R	ATCATAGCCACTGACACGGG
has_circ_0084601‐ F	GAGATGAAGGCCTTGCAGTG
has_circ_0084601‐ R	GTGTCACACTCAGCACCTTG
has_circ_0000091‐ F	GAACCAGCTGTTTACCAGAGT
has_circ_0000091‐ R	TGGTTCTGGGAACTTGAGTCT
chr14:81209418‐81329217:‐ F	TCAACAGATAGAAAGGCAGGAG
chr14:81209418‐81329217:‐ R	CGCTGGATGATTCTGCCATT
has_circ_0004249‐ F	ACAGCCAAAGGGGACTATCC
has_circ_0004249‐ R	CCAAGGAGCCACCATTTTCC
FOXP1‐ F	TGACAAACAACCAGCTCTTCA
FOXP1‐ R	TGAGGGCTCAGCACTTGTT
OCN‐ F	TCACACTCCTCGCCCTATTG
OCN‐ R	GCCTGGGTCTCTTCACTACC
RUNX2‐F	TCGCCTCACAAACAACCACA
RUNX2‐R	GACTCTGTTGGTCTCGGTGG
GAPDH‐ F	TCGGAGTCAACGGATTTGGT
GAPDH‐ R	TTCCCGTTCTCAGCCTTGAC
U6‐ F	CTCGCTTCGGCAGCACA
U6‐ R	AACGCTTCACGAATTTGCGT
miR‐33a‐5p‐ F	CCTCATAAGCGGTGCATTGTA
miR‐33a‐5p‐ R	TATGCTTGTTCTCGTCTCTGTGTC

### 2.4 Dual‐luciferase reporter assay

PCR was used to amplify circFOXP1 or FOXP1 3′‐UTR fragments containing wild‐type (WT) or mutated (MUT) predicted potential miR‐33a‐5p binding sites. They were then cloned into the psiCHECK‐2 vector (Promega Corp., Madison, WI, USA) to form a WT or MUT luciferase reporter plasmid. HEK293T cells were cultured in 24‐well plates and cotransfected with 100‐nmol/L miR‐33a‐5p mimics or negative control (miR‐NC), 1 μg of MUT or WT luciferase reporter plasmid and Lipofectamine 3000 (Invitrogen). Two days later, dual‐luciferase reporter assay system (Promega) was used to explore luciferase activity following a standard procedure. Luciferase activity was standardized using Renilla luciferase and expressed relative to basal activity.

### 2.5 RNA‐binding protein immunoprecipitation (RIP) assay

RIP assay was performed using the Magna RIP Kit (Millipore, USA) and Ago2 antibody (Cell Signaling Technology, USA) following a standard procedure. The transfected cells (2 × 10^7^) were washed in ice‐cold PBS twice and lysed in the same volume of RIP lysis buffer. Then, lysates were incubated with 5 μg of anti‐Argonaute‐2 (AGO2) antibody or non‐specific anti‐IgG antibody (Millipore) for 2 hours at 4°C. Subsequently, 50 μL of prepared magnetic beads were added to the cell lysates and incubated at 4°C overnight. The beads were then washed with RIP buffer five times and resuspended in 500 μL of TRIzol LS (Life Technology, USA) to obtain enriched RNA. Purified RNA was used for qRT‐PCR.

### 2.6 Cell culture and osteogenic differentiation induction

The hASCs were extracted from three different donors at the ScienCell Research Laboratory (Carlsbad, CA, USA). The cells were cultured in proliferation medium (PM) consisting of Dulbecco's modified Eagle's medium (Gibco, USA), 10% (v/v) foetal bovine serum (ScienCell) and 1% (v/v) penicillin/streptomycin (Gibco). For osteogenic differentiation induction, hASCs were seeded at a density of 5 × 10^5^ cells/well in a 12‐well plate. When the cells reached 80% confluence, the culture medium was replaced with osteogenic medium (OM), which consisted of PM, 10‐mmol/L β‐glycerophosphate, 100‐nmol/L dexamethasone and 0.2‐mmol/L L‐ascorbic acid (Sigma‐Aldrich). The cells were incubated under 5% CO_2_ atmosphere and full relative humidity at 37°C.

### 2.7 Cell transfection

The circFOXP1‐overexpressed or FOXP1‐overexpressed and mock pcDNA3.1 plasmids, small interfering RNAs (siRNAs) targeting circFOXP1 or FOXP1 and non‐specific negative control oligos (si‐control), miR‐33a‐5p mimic, inhibitor and negative control were purchased from GenePharma (Shanghai, China). The hASCs were seeded in six‐well plates one day prior to the transfection at 50‐60% confluence. They were then transfected with Lipofectamine 3000 (Invitrogen, Carlsbad, CA, USA) following manufacturer instructions.

### 2.8 Alkaline phosphatase staining and quantification

The hASCs were cultured in OM or PM for one week and evaluated using alkaline phosphatase (ALP) staining and quantification. ALP staining was performed with an NBT/BCIP staining kit (Beyotime Biotechnology, Shanghai, China). ALP assay kit (Nanjing Jiancheng Bioengineering Institute, Nanjing, China) was used to detect the ALP concentration. Total protein content was measured using the Pierce BCA protein assay kit (Thermo Fisher Scientific, Rockford, IL). ALP activity was calculated relative to the control group by standardizing samples to the total protein content.

### 2.9 Alizarin red S (ARS) staining and quantification

The hASCs were cultured in OM or PM for two weeks and then used for the mineralization assay. After fixation in 95% ethanol, the cells were stained with the 1% ARS staining solution (pH 4.2; Sigma‐Aldrich) for 20 minutes at room temperature. To quantify mineralization levels, the stains were dissolved using 100‐mmol/L cetylpyridinium chloride (Sigma‐Aldrich) for 1 hour and measured at 562 nm with an EnSpire multimode plate reader (PerkinElmer, Waltham, MA). Relative ARS intensity was quantified after samples were standardized to the total protein content.

### 2.10 Western blotting

Total protein samples were extracted using a radioimmunoprecipitation assay (RIPA) lysis buffer (Beyotime Biotechnology, Nantong, China) containing 1% protease inhibitor (Cell Signaling Technology). A BCA Protein Assay Kit (Beyotime Biotechnology, Nantong, China) was used to detect protein concentrations. Total protein samples (20 μg) were separated using 10% sodium dodecyl sulphate polyacrylamide gel electrophoresis (SDS‐PAGE) and subsequently transferred to polyvinylidene difluoride (PVDF) membranes. The membranes were incubated with primary antibodies specific for anti‐FOXP1 (ab134055, 1:1000), anti‐OCN (ab93876, 1:500), anti‐RUNX2 (ab23981, 1 μg/mL) and anti‐GAPDH (ab9485, 1:2500) (Abcam, Cambridge, UK, USA) at 4°C overnight. They were then incubated with horseradish peroxidase‐labelled secondary antibodies (Santa Cruz, Dallas, TX, USA) for 2 hours at room temperature. An enhanced chemiluminescence (ECL) kit (Solarbio, China) was used to visualize protein bands.

### 2.11 In vivo heterotopic bone formation assay

Female BALB/C homozygous nude (nu/nu) mice (aged six weeks, n = 42) were obtained from the Laboratory Animal Center of Zhejiang Chinese Medical University and maintained under specific pathogen‐free conditions in a 12‐hours light/dark cycle with water and food provided ad libitum. Institutional Animal Care and Use Committee of Zhoushan Hospital of Traditional Chinese Medicine Affiliated to Zhejiang Chinese Medical University approved this study. All animal experiments were carried out following the institutional and national guidelines.

The hASCs transfected with circFOXP1 overexpression vector and/or miR‐33a‐5p mimics, si‐circFOXP1 and/or miR‐33a‐5p inhibitor, or non‐transfected cells (NC) were cultured in PM for one week prior to in vivo analysis. The cells (1 × 10^7^ cells/mL) were then collected and resuspended in 15 μL of Col‐Tgel component A and 1.5 μL of component B (Bioruo, Beijing, China). The hASCs encapsulated in collagen‐based hydrogels were implanted into dorsal subcutaneous pockets of nude mice (six mice/group). After two months of implantation, the samples were harvested and some were frozen to perform qRT‐PCR and Western blot analyses. The remaining tissue samples were fixed in 10% neutral formaldehyde fixative and decalcified in 10% ethylenediaminetetraacetic acid (pH 7.4) for two weeks. The specimens were sectioned into 4‐µm‐thick paraffin‐embedded sections and then analysed using haematoxylin and eosin (HE), Masson's and IHC staining with primary antibodies against osteocalcin (OCN; Abcam) to detect osteogenesis.

### 2.12 Immunohistochemistry (IHC) assay

The slices (4‐μm‐thick) were dewaxed and rehydrated using xylene and ethanol, and high‐pressure heat was applied for antigen retrieval. After blocking with goat serum, the sections were incubated with the first antibody against OCN (ab93876, 5 µg/mL, Abcam) overnight at 4°C. The sections were then incubated with HRP‐conjugated secondary antibodies at room temperature. The immunoreactive signals were detected with a diaminobenzidine (DAB) detection kit (ZSGB‐BIO, Beijing, China). Finally, the tissue slices were observed and photographed under a light microscope (Olympus, Tokyo, Japan).

### 2.13 Statistical analysis

Statistical analysis was performed using SPSS (IBM, Armonk, NY, USA) and Prism (GraphPad Software, La Jolla, CA, USA) software. Quantitative results were expressed as mean ± standard deviation based on at least three independent experiments per group. Independent two‐tailed Student's *t* tests were used to compare groups. Tukey's post hoc test and one‐way ANOVA were used for comparisons among multiple groups. Correlations were calculated using Pearson's correlation analysis. *P*‐values < 0.05 denoted statistical significance.

## 3 RESULTS

### 3.1 Expression profile for circRNA in OP patients

**FIGURE 1:**
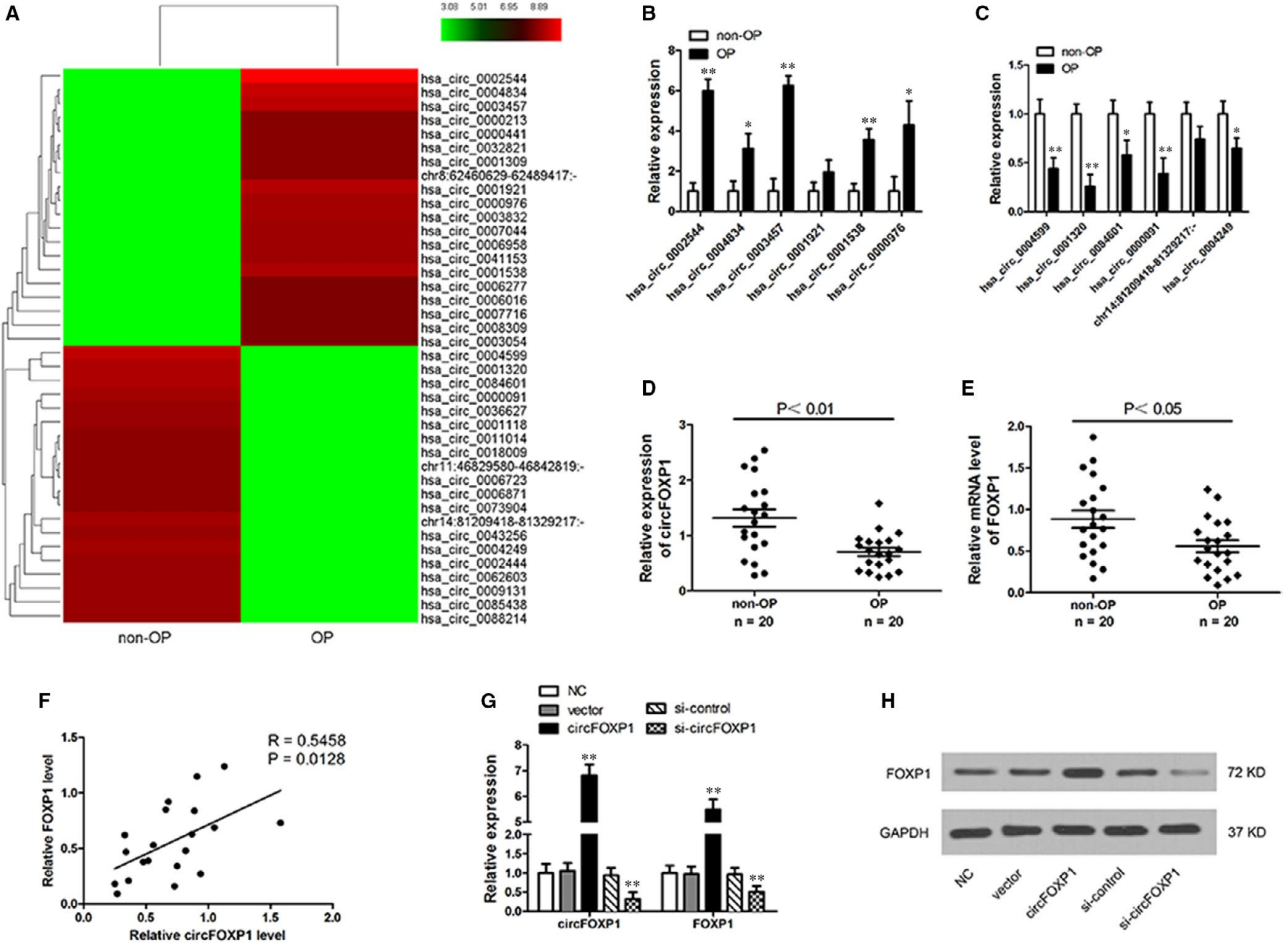
CircRNA expression profiles in OP patients. A, Cluster heat map for top 40 differentially expressed circRNAs (20 up‐regulated and 20 down‐regulated) in OP and non‐OP bone tissues. Red colour represents high expression level, green colour represents low expression level. B and C, Relative expression for six most up‐regulated (B) and six most down‐regulated (C) circRNAs validated by qRT‐PCR. D, Relative expression of circFOXP1 in OP (n = 20) and non‐OP (n = 20) bone tissues determined by qRT‐PCR. E, FOXP1 mRNA expression level in OP (n = 20) and non‐OP (n = 20) bone tissues. F, CircFOXP1 is positively correlated with FOXP1 mRNA in OP tissues. G and H, FOXP1 mRNA (G) and protein (H) levels determined by qRT‐PCR and Western blotting in hASCs with circFOXP1 overexpression or knockdown. All experiments were performed at least three times. Untreated hASCs were used as negative control (NC). **P < 0.01 vs NC group. OP, osteoporosis; hASCs, human adipose‐derived stem cells

To detect potential circRNA roles in OP, circRNA microarray analyses were conducted in OP and non‐OP bone tissues (n = 20/group). It was discovered that 2327 circRNAs were down‐regulated in OP tissues with a fold difference of >2 (*P* < 0.05). A total of 2645 circRNAs were up‐regulated using the same cut‐off. Hierarchical clustering illustrated top 20 down‐regulated or up‐regulated circRNAs in OP tissues (Figure 1A). Subsequently, a subset of top differentially expressed circRNAs (the six most up‐regulated and the six most down‐regulated) were chosen for verification by qRT‐PCR (Figure 1B,C). The qRT‐PCR analysis demonstrated that has_circ_0001320, a circRNA originating from the FOXP1 gene (a.k.a. circFOXP1), was the most significantly regulated circRNA among the verified top down‐regulated circRNAs in OP patient bone tissues compared to non‐OP bone tissues (Figure 1C,D).

Recent studies have discovered that circRNAs regulate corresponding linear transcript function and expression.^33^, ^34^ In this manner, regulatory correlation between circFOXP1 and its linear transcript (FOXP1) was explored in the present investigation. FOXP1 expression was found to be significantly down‐regulated in OP bone tissues compared to non‐OP bone tissues (Figure 1E). Pearson's correlation analysis revealed a significant positive correlation between circFOXP1 and FOXP1 in OP bone tissues (Figure 1F). Moreover, FOXP1 mRNA and protein levels were significantly up‐regulated or down‐regulated when circFOXP1 expression was artificially altered in hASCs with circFOXP1 overexpression or knockdown, respectively (Figure 1G,H). These results suggest that FOXP1 may be a circFOXP1 target gene.

### 3.2 CircFOXP1 functions as miR‐33a‐5p sponge

**FIGURE 2:**
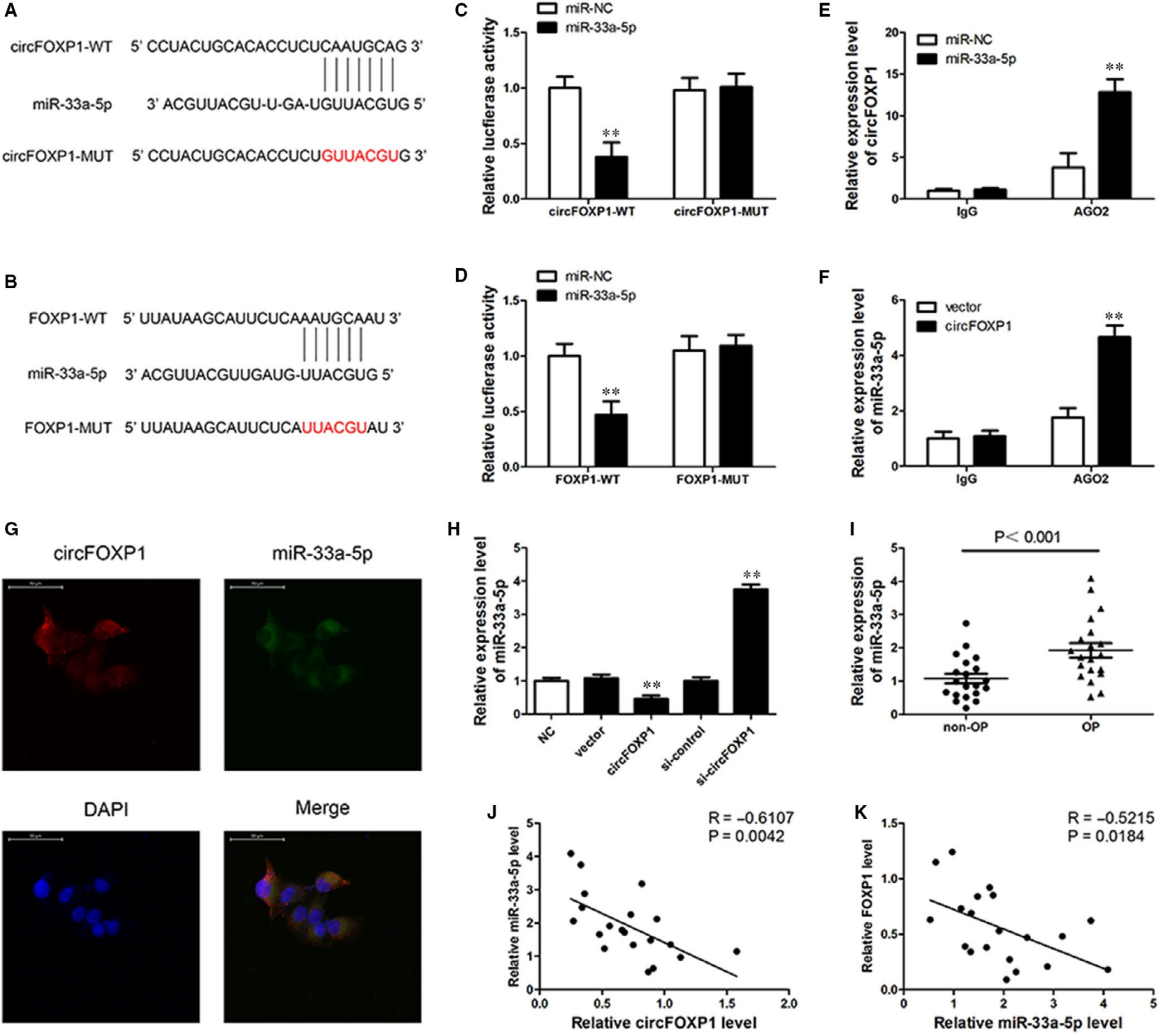
CircFOXP1 serves as miR‐33a‐5p sponge. A and B, Putative binding site for miR‐33a‐5p in circFOXP1 and FOXP1 3′‐UTR. Red colour indicates mutated miR‐506‐3p‐binding site sequence. C and D, Relative luciferase activities analysed in 293T cells cotransfected with miR‐33a‐5p mimics or control miRNA (miR‐NC) and WT or MUT luciferase reporter containing circMID1 (C) or FOXP1 3′‐UTR (D, **P < 0.01). E, AGO2 RNA immunoprecipitation (RIP) assay for circFOXP1 levels in hASCs transfected with miR‐33a‐5p or miR‐NC (**P < 0.01). F, Ago2 RIP assay for miR‐33a‐5p levels in hASCs overexpressing circFOXP1 or vector control (**P < 0.01). G, Fluorescence in situ hybridization assay conducted to determine co‐localization between circFOXP1 and miR‐33a‐5p. Scale bar = 50 μm. H, MiR‐33a‐5p expression level determined by RT‐PCR in hASCs with vector or circFOXP1 overexpression, si‐control, or si‐circFOXP1. Untreated hASCs were used as negative control (NC). **P < 0.01 vs NC group. I, Relative expression for miR‐33a‐5p in OP (n = 20) and non‐OP (n = 20) bone tissues determined by qRT‐PCR. J, Pearson correlation analysis shows negative correlation between miR‐33a‐5p and circFOXP1 (*R* = −0.6107; *P* = 0.0042) or FOXP1 (*R* = −0.5215; *P* = 0.0184) in OP tissues. hASCs, human adipose‐derived stem cells; OP, osteoporosis

Given that circRNAs act as an miRNA sponge to regulate gene expression, potential miRNA associated with circFOXP1 and FOXP1 targeting was predicted using bioinformatics analysis. Among these target miRNAs, circFOXP1 and FOXP1 had a binding site for miR‐33a‐5p, and the predicted miR‐33a‐5p binding site was mutated (Figure 2A,B). To ensure miR‐33a‐5p and circFOXP1/FOXP1 target relationships, luciferase reporter plasmids with MUT or WT circFOXP1 or FOXP1 sequence were cotransfected with miR‐33a‐5p mimics or negative control. The luciferase activity was significantly lower in the miR‐33a‐5p mimic group cotransfected with WT circFOXP1 or WT FOXP1 3′UTR (Figure 2C,D). RIP assay was performed to pull down RNA transcripts bound to Ago2 in hASCs. The circFOXP1 and miR‐33a‐5p were pulled down efficiently by anti‐Ago2, but not by the non‐specific anti‐IgG antibody (Figure 2E,F). Moreover, RNA FISH assay results demonstrated that circFOXP1 and miR‐33a‐5p were co‐located in the cytoplasm (Figure 2G). Overexpression or silencing of circFOXP1 reduced or enhanced miR‐33a‐5p expression, respectively (Figure 2H). In addition, miR‐33a‐5p expression levels in the OP samples were much lower than those in the non‐OP bone tissues (Figure 2I). Furthermore, miR‐33a‐5p was negatively correlated with circFOXP1 or FOXP1 in OP bone tissues (Figure 2J,K). These findings confirm that circFOXP1 can function as an miR‐33a‐5p sponge to regulate FOXP1 expression.

### 3.3 CircFOXP1 regulates hASC osteogenic differentiation in vitro via circFOXP1/miR‐33a‐5p/FOXP1 pathway

**FIGURE 3:**
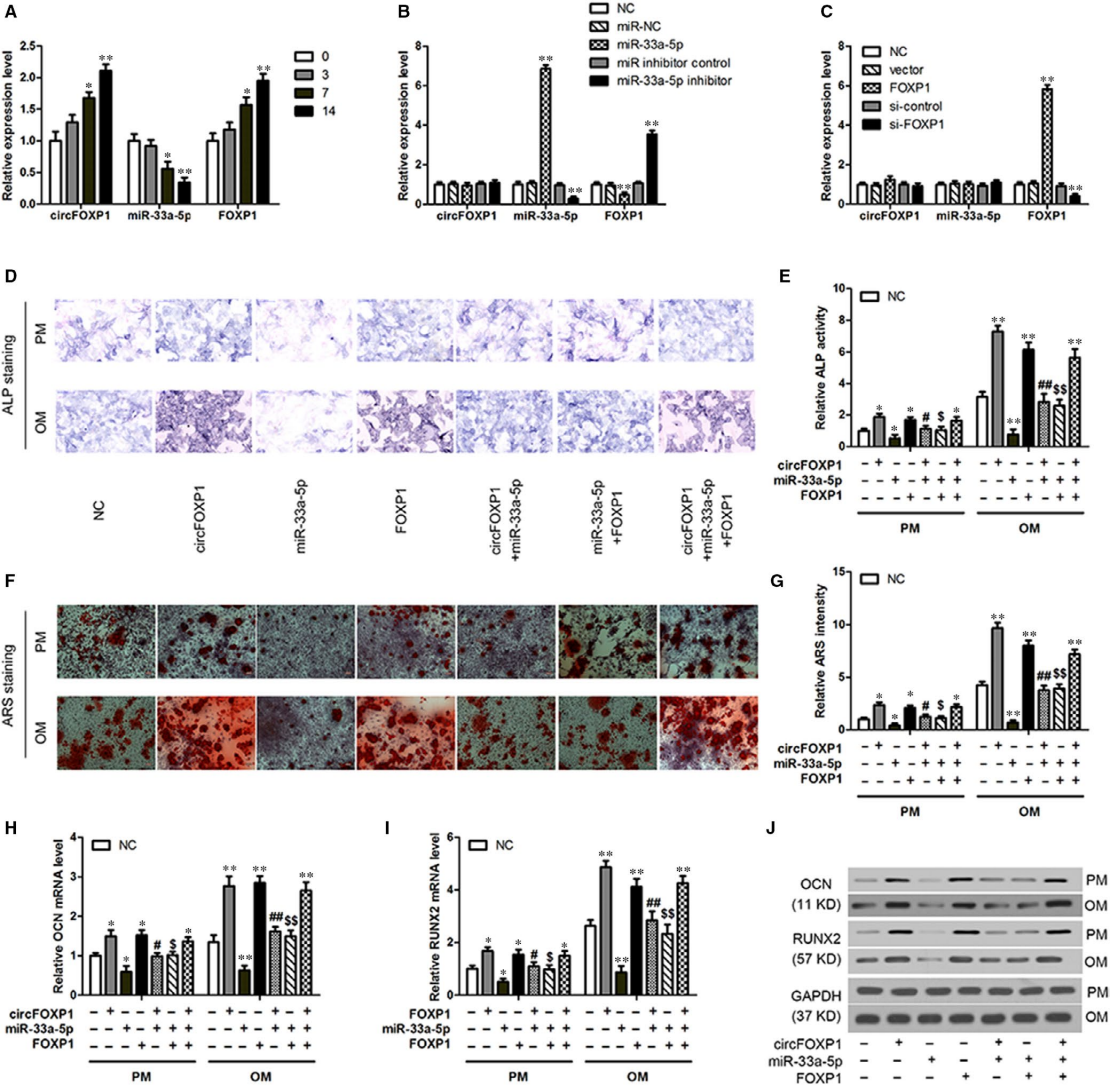
CircFOXP1 overexpression promotes in vitro osteogenic differentiation in hASCs by regulating miR‐33a‐5p/FOXP1. A, QRT‐PCR analysis of circFOXP1, miR‐33a‐5p and FOXP1 expression during osteogenic differentiation in hASCs at different time points. *P < 0.05, **P < 0.01 vs 0 d time point. B and C, Relative expression of circFOXP1, miR‐33a‐5p and FOXP1 in hASCs transfected with miR‐33a‐5p mimics or inhibitor (B) or with FOXP1 overexpression or knockdown (C). **P < 0.01 vs NC group. HASCs were transfected with circFOXP1, miR‐33a‐5p mimics, and/or FOXP1 overexpression. D, ALP staining of transduced hASCs cultured in PM or OM for seven days (scale bar = 200 μm). E, ALP quantification on day 7 in PM and OM. F, ARS staining of transduced hASCs cultured in PM or OM for 14 d (scale bar = 100 μm). G, ARS quantification on day 14 in PM and OM. H and I, Relative mRNA expression of RUNX2 and OCN on day 7 in transduced hASCs cultured in PM or OM was measured by qRT‐PCR. J, Western blot analysis detected RUNX2 and OCN protein expression levels on day 7 in transduced hASCs cultured in PM or OM. Untreated hASCs were used as negative control (NC). *^, #, $^ *P* < 0.01, **^, ##, $$^ *P* < 0.01, *vs NC group, ^#^vs circFOXP1 group, ^$^vs miR‐33a‐5p group. ALP, alkaline phosphatase; ARS, alizarin red S; hASCs, human adipose‐derived stem cells; OCN, osteocalcin; OM, osteogenic medium; PM, proliferative medium; RUNX2, runt‐related transcription factor 2

To detect the circFOXP1/miR‐33a‐5p/FOXP1 axis involvement in hASC osteogenic differentiation, circFOXP1, miR‐33a‐5p and FOXP1 expression levels were examined after 0, 3, 7 and 14 days of osteogenic differentiation. During hASC osteogenic differentiation, circFOXP1 and FOXP1 expression levels tended to increase, whereas miR‐33a‐5p expression decreased gradually (Figure 3A). Overexpression of circFOXP1 reduced miR‐33a‐5p and enhanced FOXP1 mRNA, whereas circFOXP1 knockdown led to the opposite results (Figures 1E and 2H). Moreover, FOXP1 expression was up‐regulated or down‐regulated by miR‐33a‐5p mimics or inhibitors in hASCs, respectively, whereas circFOXP1 expression remained unchanged (Figure 3B). However, circFOXP1 and miR‐33a‐5p expression levels were not regulated by FOXP1 (Figure 3C).

To evaluate circFOXP1, miR‐33a‐5p and FOXP1 roles in hASC osteogenesis, these genes were overexpressed or inhibited in hASCs. ALP and ARS staining was used to predict the mineralization level in vitro. Compared to the NC group, ALP staining and activity on day 7 in hASCs with circFOXP1 or FOXP1 overexpression were significantly enhanced. The opposite results were discovered in hASCs with miR‐33a‐5p mimics in PM and OM (Figure 3D,E). In addition, ARS staining and quantification after 14 days suggested that the extracellular matrix mineralization was significantly elevated by circFOXP1 or FOXP1 overexpression and significantly inhibited by miR‐33a‐5p mimics in hASCs in OM and PM, respectively (Figure 3F,G). Consistently, mRNA expression of osteogenesis‐related genes, such as OCN and RUNX2, was up‐regulated by circFOXP1 or FOXP1 overexpression and down‐regulated by miR‐33a‐5p mimics compared to the NC group in hASCs in PM and OM (Figure 3H,I). Furthermore, RUNX2 and OCN protein expression generated similar results compared to those obtained in the qRT‐PCR analysis (Figure 3J).

**FIGURE 4:**
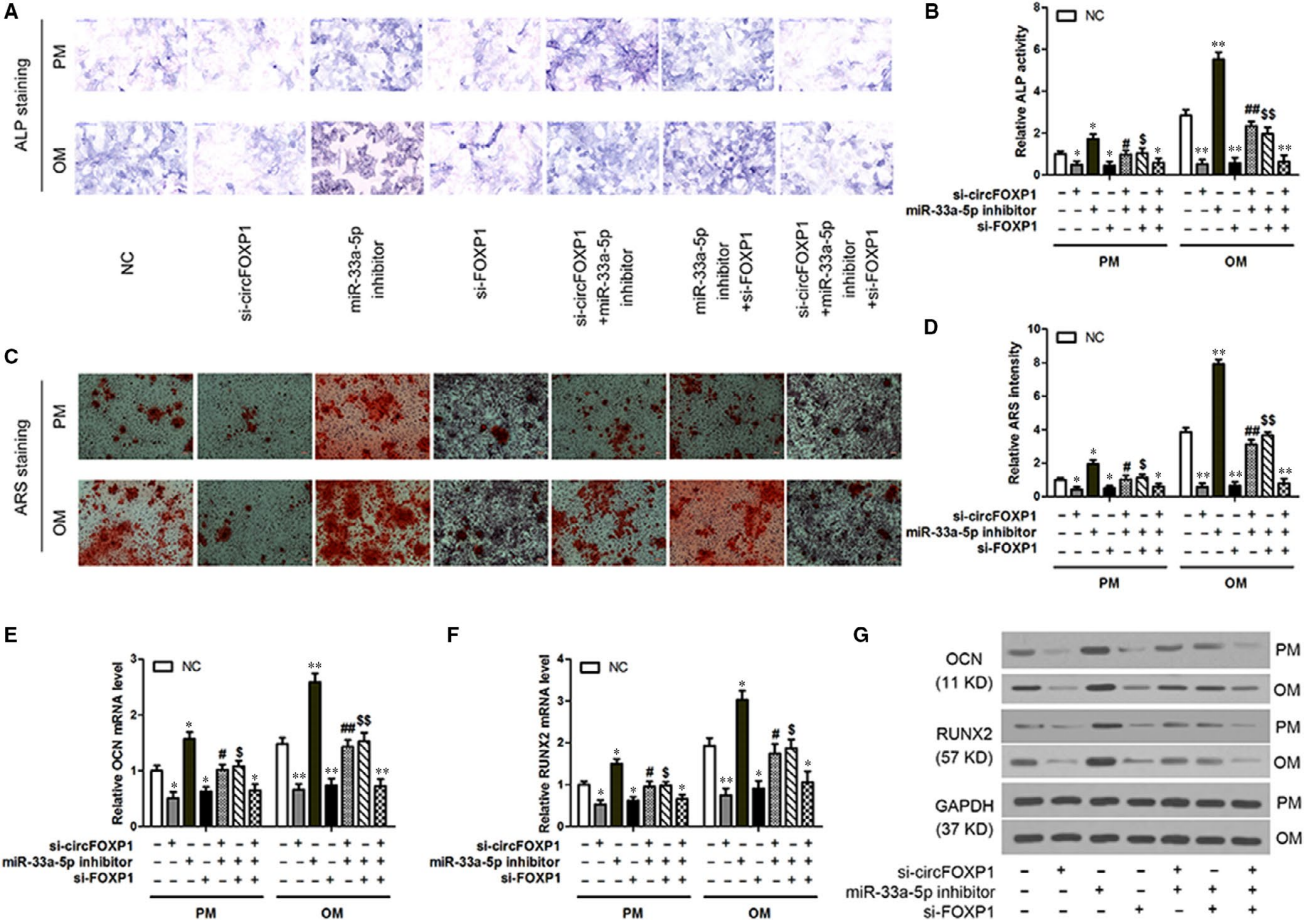
CircFOXP1 knockdown inhibits in vitro osteogenic differentiation in hASCs by regulating miR‐33a‐5p/FOXP1. HASCs were transfected with si‐circFOXP1, miR‐33a‐5p inhibitor and/or si‐FOXP1. A, ALP staining of transduced hASCs cultured in PM or OM for seven days (scale bar = 200 μm). B, ALP quantification on day 7 in PM and OM. C, ARS staining of transduced hASCs cultured in PM or OM for 14 d (scale bar = 100 μm). D, ARS quantification on day 14 in PM and OM. E and F, Relative mRNA expression of RUNX2 and OCN on day 7 in transduced hASCs cultured in PM or OM measured by qRT‐PCR. G, Western blot analysis detected RUNX2 and OCN protein expression levels on day 7 in transduced hASCs cultured in PM or OM. Untreated hASCs were used as negative control (NC). *^, #, $^ *P* < 0.01, **^, ##, $$^ *P* < 0.01, *vs NC group, ^#^vs circFOXP1 group, ^$^vs miR‐33a‐5p group. ALP, alkaline phosphatase; ARS, alizarin red S; hASCs, human adipose‐derived stem cells; OCN, osteocalcin; OM, osteogenic medium; PM, proliferative medium; RUNX2, runt‐related transcription factor 2

To validate the role of circFOXP1/miR‐33a‐5p/FOXP1 in osteogenic differentiation, hASCs transfected with circFOXP1 siRNA (si‐circFOXP), miR‐33a‐5p inhibitor or FOXP1 siRNA (si‐FOXP) were cultured in PM or OM. The osteogenic differentiation of hASCs was significantly inhibited by si‐circFOXP1 or si‐FOXP1 and enhanced by the miR‐33a‐5p inhibitor compared to the NC group, as demonstrated by the ALP (Figure 4A,B) and ARS (Figure 4C,D) staining and quantification and RUNX2 and OCN (Figure 4E‐G) expression results. The effect of circFOXP1/FOXP1 overexpression or knockdown in the above experiments was eliminated by miR‐33a‐5p mimics or inhibitors in cotransfected hASCs, respectively. These demonstrated the role of circFOXP1 in hASC osteogenic differentiation in vitro.

### 3.4 CircFOXP1 and miR‐33a‐5p affect hASC osteogenic differentiation in vivo by regulating FOXP1

To further confirm that circFOXP1 osteogenesis regulation in vivo is similar to that in vitro, hASCs with circFOXP1 overexpression and/or miR‐33a‐5p mimics were encapsulated in collagen‐based hydrogels and implanted into the nude mouse dorsal subcutaneous space. Implantation samples were extracted after eight weeks for analysis.

**FIGURE 5:**
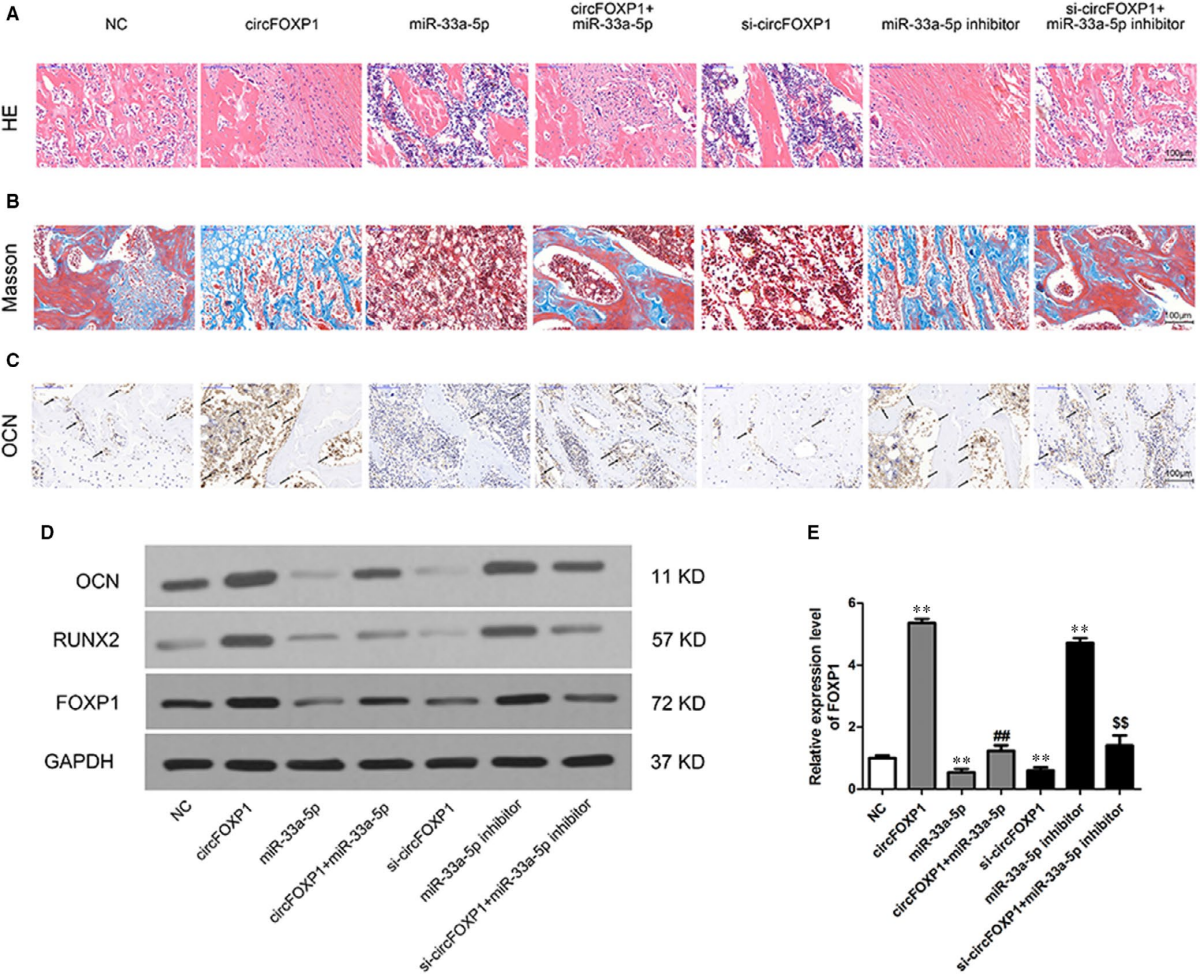
CircFOXP1 and miR‐33a‐5p affect in vivo osteogenic differentiation of hASCs by regulating FOXP1. A‐C, Histological assessment of ectopic bone formation (scale bar = 100 μm): A, HE; B, Masson's and C, IHC staining for OCN. Dark brown granules in cells denote OCN‐positive staining (partially marked with black arrows). D, Western blot analysis for OCN, RUNX2 and FOXP1 protein expression in implantation samples. E, Relative FOXP1 mRNA expression in implantation samples. *^, #, $^ *P* < 0.01, **^, ##, $$^ *P* < 0.01, *vs NC group, ^#^vs circFOXP1 group, ^$^vs si‐circFOXP1 group. hASCs, human adipose‐derived stem cells; HE, haematoxylin and eosin; IHC, immunohistochemical; OCN, osteocalcin

HE staining revealed more newly constructed bone in the circFOXP1 or miR‐33a‐5p inhibitor group and less osteoid tissue in the miR‐33a‐5p or si‐circFOXP1 group compared to the other three groups (Figure 5A). Masson's trichrome staining demonstrated more collagen fibre bundles arranged compactly in the circFOXP1 or miR‐33a‐5p inhibitor group and less collagen organization (in blue) in the miR‐33a‐5p or si‐circFOXP1 group compared to the other three groups (Figure 5B). Furthermore, IHC staining for OCN revealed a significant amount of brown stained granules widely distributed in hASCs in the circFOXP1 or miR‐33a‐5p inhibitor group. The miR‐33a‐5p or si‐circFOXP1 group contained fewer stained granules in the cells compared to the other three groups (Figure 5C). Western blot analysis for the OCN and RUNX2 expression levels revealed similar results (Figure 5D). There were no significant differences between the circFOXP1 + miR‐33a‐5p or si‐circFOXP1 + miR‐33a‐5p inhibitor group and the NC group, implying that miR‐33a‐5p can eliminate the circFOXP1 effect on osteogenic differentiation in hASCs in vivo (Figure 5A‐D). The qRT‐PCR and Western blot analysis results showed that FOXP1 expression level was markedly increased by the circFOXP1 or miR‐33a‐5p inhibitor and decreased by the miR‐33a‐5p or si‐circFOXP1. It remained unchanged in the circFOXP1 + miR‐33a‐5p or si‐circFOXP1 + miR‐33a‐5p inhibitor group compared to the NC group (Figure 5D,E). In summary, circFOXP1/miR‐33a‐5p was able to regulate hASC osteogenesis in vivo by regulating FOXP1.

## 4 DISCUSSION

OP is a progressive metabolic skeletal condition that increases the fragility fracture risk caused by low bone mass architectural deterioration of bone microstructure because of an imbalance between bone resorption and bone formation.^35^, ^36^, ^37^ To date, substantial investigations have reported that circRNAs take part in regulating different pathophysiological events, including tumorigenesis, organogenesis and tissue development, and function as biomarkers in various human processes, such as osteoporosis and cancer.^38^, ^39^, ^40^ In the present study, circRNA expression profiles were evaluated and 4972 circRNAs were significantly expressed in the OP group compared to the non‐OP group. Among differentially expressed circRNAs, circFOXP1 had a lower expression in OP patients and was up‐regulated during osteogenic differentiation in hASCs. Further experiments showed that circFOXP1 enhances the osteogenic differentiation in vitro and hASC osteogenesis in vivo. Recently, Cherubini et al reported that circFOXP1 promotes MSC differentiation and proliferation by sponging miR‐17‐3p and miR‐127‐5p, suggesting its key role in stem cell fate decision‐making processes.^32^ These consistent findings reveal that circFOXP1 might have a vital function in osteogenesis and may be a therapeutic OP target.

It is notable that expression of the corresponding FOXP1 linear transcripts was also decreased and positively correlated with circFOXP1 expression in OP. Moreover, the regulatory relationship between circFOXP1 and FOXP1 was first confirmed in our subsequent experiments, showing that circFOXP1 regulates the FOXP1 expression by acting as an miRNA sponge. In a previous study, FOXP1 haploinsufficiency resulted in craniofacial structure deformity and speech ability defect.^41^ FOXP1 was also identified as a dose‐dependent orchestrator of MSC senescence and differentiation potency in skeletal ageing.^31^ Most importantly, FOXP1 or circFOXP1 overexpression was correlated with many cancers.^42^, ^43^, ^44^, ^45^ The multiple roles of FOXP1 and its circRNA involvement in MSC plasticity and disease progression regulation make them as the potential anabolic targets for OP therapy.

Prior studies have reported that circRNAs are generated by splicing during pre‐mRNA maturity and some of them might function as miRNA sponges.^46^ The present study illustrates that circFOXP1 can bind to miR‐33a‐5p and that miRNA negatively regulates hASC osteogenic differentiation. The latest research findings suggest that miR‐33a‐5p and 3p regulate hMSC osteoblast differentiation by targeting EGFR and YAP signalling.^26^ The present study has shown that miR‐33a‐5p is up‐regulated in OP, although its expression is gradually decreased during hASC osteogenic differentiation. However, Mi et al^47^ discovered that miR‐33a‐5p expression is up‐regulated with TNF‐α treatment in BMP‐2‐induced hBMSC osteogenic differentiation. These conflicting findings may be explained by the differences in cell sample sources and different treatments.

MSCs are a good cell source for cell‐based therapy for various conditions, including OP, because of their self‐renewal properties, multilineage differentiation potential and low immunogenicity.^48^, ^49^ Extensive investigations have shown that biomaterial scaffolds loaded with bone marrow‐derived MSCs (BMSCs) enhance bone regeneration and cartilage repair.^50^, ^51^, ^52^ When comparing to BMSCs, osteogenic proliferation and differentiation of adipose‐derived MSCs (ASCs) are less affected by multiple passage and age, making ASCs a candidate source for cell‐based therapy, particularly in elderly OP patients.^53^ Mirsaidi et al evaluated the therapeutic effects of ASCs in OP senescence‐accelerated mice (SAMP6) and suggested ASCs for an autologous cell‐based approach to treat OP.^54^ The in vivo experiments in the present study support the fact that circFOXP1 can promote hASC heterotopic bone formation by regulating the miR‐33a‐5p/FOXP1 pathway. The preclinical studies with animal models have demonstrated that ASCs are effective in treating osteoporosis. One phase II clinical trial using hASCs for treatment of proximal humeral fracture as a model for osteoporotic fracture has been conducted by University Hospital in Basel, Switzerland. (available online: http://clinicaltrials.gov, NCT01532076). The hASCs were isolated from the patient, seeded within a composite graft and transplanted back into the fracture site. The clinical/radiological follow‐up and functional assessment were performed. However, the trial was terminated and no results were reported at present. Therefore, more preclinical and clinical studies are needed to determine the therapeutic effects of ASCs for treating osteoporosis.

## 5 CONCLUSION

In this study, circFOXP1 was down‐regulated in OP. Study results demonstrated that circFOXP1 might promote hASC osteogenesis in vitro and in vivo by targeting the miR‐33a‐5p/FOXP1 pathway. Taken together, these data suggest that circFOXP1 may regulate osteogenesis and bone regeneration and may be a candidate target for hASC‐based therapy for OP treatment.

## CONFLICT OF INTEREST

None declared.

## AUTHOR CONTRIBUTIONS

**Wanxiang Shen:** Conceptualization (equal); Data curation (equal); Formal analysis (lead); Resources (equal); Writing‐original draft (equal). **Bin Sun:** Formal analysis (equal); Investigation (equal); Methodology (equal); Software (equal). **Chenghong Zhou:** Data curation (equal); Formal analysis (supporting); Methodology (equal). **Wenyi Ming:** Data curation (equal); Investigation (equal); Visualization (equal). **Shaohua Zhang:** Investigation (equal); Software (equal); Visualization (supporting). **Xudong Wu:** Conceptualization (equal); Funding acquisition (lead); Project administration (lead); Resources (equal); Supervision (equal); Validation (equal); Writing‐original draft (supporting); Writing‐review & editing (lead).

## Data Availability

The datasets generated for this study are available on request to the corresponding author.
